# Canjiqueira Fruit: Are We Losing the Best of It?

**DOI:** 10.3390/foods9040521

**Published:** 2020-04-21

**Authors:** Daniela G. Arakaki, Vanessa Samúdio dos Santos, Elaine Pádua de Melo, Hugo Pereira, Priscila Silva Figueiredo, Mário Rodrigues Cortês, Carlos Alexandre Carollo, Lincoln Carlos Silva de Oliveira, Paula Tschinkel, Francisco Reis, Igor Souza, Rafaela Rosa, Fabiane Sanches, Elisvânia Freitas dos Santos, Valter Aragão do Nascimento

**Affiliations:** 1 Graduate Program in Health and Development in the Midwest Region of Brazil, Federal University of Mato Grosso do Sul, 79070-900 Campo Grande, Brazil; elainespmelo@hotmail.com (E.P.d.M.); hugo.23x@gmail.com (H.P.); prifigueiredo92@gmail.com (P.S.F.); paulasaldanhatschinkel@gmail.com (P.T.); fjmreis@uol.com.br (F.R.); igor_domingos_souza@hotmail.com (I.S.); nutricionista.rafaelarosa@gmail.com (R.R.); elisvania@gmail.com (E.F.d.S.); 2 Group of Spectroscopy and Bioinformatics Applied Biodiversity and Health (GEBABS), Federal University of Mato Grosso do Sul, 79070-900 Campo Grande, Brazil; 3 Laboratory of Natural Products and Mass Spectrometry, Federal University of Mato Grosso do Sul, 79070-900 Campo Grande, Brazil; vanessamudy@yahoo.com.br (V.S.d.S.); carlos.carollo@gmail.com (C.A.C.); 4 Chemistry Institute, Federal Universityof Mato Grosso do Sul, 79070-900 Campo Grande, Brazil; mariocortess40@gmail.com (M.R.C.); lincoln.oliveira@ufms.br (L.C.S.d.O.); 5 Faculty of Pharmaceutical Sciences, Food and Nutrition, Federal University of Mato Grosso do Sul, 79070-900 Campo Grande, Brazil; fabianelaflor@gmail.com

**Keywords:** byproducts, secondary metabolites, elemental content, fatty acid profile, thermal stability

## Abstract

Fruits and byproducts are valuable sources of nutrients and bioactive compounds, which are associated with a decreased risk of developing several diseases, such as cancer, inflammation, cardiovascular diseases, and Alzheimer’s. The fruits of canjiqueira (*Byrsonima cydoniifolia*) are already exploited as a food resource, while the seeds are discarded. This study aimed at showing the potential of the whole fruit of canjiqueira. Elemental characterization was performed on ICP OES, while thermal stability was assessed on thermogravimetry. The determination of the fatty acid profile was carried out on gas chromatography and bioactive compound identification using liquid chromatography and mass spectrometry. Results show that both parts of canjiqueira fruit are a source of various minerals, such as Ca, Cu, Fe, K, Mg, and Mn while the seed only is a good source for Zn. Oleic and linoleic acids are the main compounds in pulp and seed. The thermal stability of seed oil is superior to pulp oil, while piceatannol concentration is higher in seed than pulp. All parts of canjiqueira fruit may be used as a strategy to address nutrition issues and are valuable ingredients to prospective food products.

## 1. Introduction

According to the World Health Organization (WHO) [1], proximate to 5.2 million deaths across the world were related to unsatisfactory fruit and vegetable intake in the year of 2013. Fruits have macronutrients, micronutrients and vitamins, and other non-nutrients, like fibers and bioactive compounds [2,3,4]. Macro- and micro-elements are involved in cellular processes, immune function, and defense against free radicals [5]. The consumption of phytochemicals, such as carotenoids and flavonoids, are related to a lower risk of developing ailments like certain types of cancer, inflammation, cardiovascular, and neurodegenerative disorders [6,7].

Byproducts of fruits are parts of the whole fruit, constituted of skins, seeds, rinds, and portions of the flesh that are not used, which are produced by various steps of the industrial course and usually are discarded [8]. Several studies have shown that food wastes could work as a source of biocompounds, with an increased scientific interest concerning their positive effects on human health [9].

Some fruit byproducts, such as seeds, are also used as a strategy for alimentation in areas of food scarcity, being available during the whole year, when dehydrated [10,11]. Some studies showed higher mineral concentration in pulp residue than in pulp itself [12,13]. Likewise, compounds of interest are present in byproducts.

The use of the fruit pulps and their byproducts can be applied to a diverse range of interests, as in nutraceutical and pharmaceutical products, food, and dietary additives, by isolating distinctive phytochemicals. This application could contribute to recovering the agro-industrial process waste, with significant industrial, economic, and environmental impact [14].

Brazil has a tropical ecosystem rich in fruits, which may contribute to food, nutrition, and health; besides, it may be a source for income generation in Brazilian rural communities [3,15]. Canjiqueira (*Byrsonima cydoniifolia* A. Juss), also known as *B. orbygniana*, is a native fruit from Central Brazil that grows exclusively in sandy soils [16]. The ripe fruits are yellow, sized 1.5 to 2 cm. The pulp is fleshy and smooth, and its consumption is usually in natura or the form of juices, ice creams, jams, and liqueur [17,18]. Even though canjiqueira is used as a food source from the rural population, this fruit remains one of the unexplored native fruits in Brazil to achieve its full potential.

As cited above, fruits and fruits byproducts contain significant amounts of nutrients and interesting biocompounds. Fruits byproducts may contain smaller amounts of digestible energy; therefore, its use on animal feed is restricted. These fruit parts are rich in phytochemicals, which diminish their use as composting, once they show antimicrobial activity. Nevertheless, these properties may be valuable in human health [19]. One of the identified biocompounds of canjiqueira is the piceatannol, which is a *trans*-stilbene, associated with beneficial effects, including anticancer activity [20] and it is commonly present in grape skins [21], passion fruit seeds [22] and bagasse [23].

Although some studies considered the canjiqueira whole fruit or pulp antioxidant potential and biocompounds [24,25,26], along with fatty acid composition and oxidative stability [24], no studies have addressed pulp and seed apart. In this study, we investigated the canjiqueira whole fruit, determining for the first-time minerals, fatty acids, oil stability, and piceatannol in both pulp and seed. A better knowledge of native fruits makes it possible to encourage their consumption [27] and their trade [28], besides their application in food products.

## 2. Materials and Methods

### 2.1. Research Area and Plant Material

The present study was conducted according to the guidelines of the Code of Brazilian law; this research was registered with the National Genetic Resource Management System and Associated Traditional Knowledge (SisGen, No. A7716EC). Fruits of *B. cydoniifolia* A. Juss were randomly collected from 30 different shrubs near of Corumbá (−19°36′14.36″, −57°02′48.90″), Mato Grosso do Sul, Midwest region of Brazil. From this collection, a sample pool was prepared and analyzed. We collected ripe fruits in December 2016 from different plants. This specimen of fruit was deposited (No 59035) in the herbarium of the Federal University of Mato Grosso do Sul (CGMS).

### 2.2. Sample Preparation

The pulp and the seed of canjiqueira were removed using plastic knives and then kept separated. The samples were dried in a circulation oven and grounded with a manual grinder into a powder. We used these samples in all the following analyses.

### 2.3. Microwave Digestion

All glassware and polyethylene bottles were kept 24 h soaking in 10% HNO_3_ and cleaned by rinsing five times with ultrapure water. Separately to each other, pulp and seed were accurately weighed on a microanalytical scale and processed with a mixture of 0.25 g sample plus 2 mL nitric acid (HNO_3_, 65% Merck) and 1 mL hydrogen peroxide (H_2_O_2_, 35% Merck). All samples were packed in vessels of Teflon of the microwave oven (Speedwave^®^ Four, Berghof, Germany). The conditions for the process of digestion of samples are available on Supplementary Material.

After samples digestion, they were diluted to 10 mL with ultrapure water (ELGA, Veolia Water Solutions & Technologies, Houston, TX, USA).

### 2.4. Elemental Analysis by Inductively Coupled Plasma Optical Emission Spectrometry (ICP OES)

The contents of macro- and micro-elements (Na, K, Ca, Mg, and Al, Co, Cu, Fe, Mo, Mn, Se, and Zn) in pulp and seed were measured by ICP OES (Thermo Scientific—iCAP 6000 Series, Waltham, MA, USA). The elemental content in these samples was determined using a standard calibration curve for each analyzed element. The best-operating conditions of ICP OES were optimized according to Supplementary Material.

### 2.5. Calibration Curves

A diluted standard solution for each of the elements Al, Ca, Co, Cu, Fe, K, Mg, Mo, Mn, Na, Se, and Zn was set from basic multielementar stock solution with 1000 mg/L (SpecSol, Quimlab, Brazil).

The sensitivity of macro- and micro-element detection was determined by the slope of the linear regression equation. The evaluation of linearity was measured by the correlation coefficients of the calibration curves. It was accepted when they were equal or above 0.9969 A twelve-point calibration curve was generated using the following concentrations: 0.001, 0.0026, 0.005, 0.01, 0.025, 0.05, 0.1, 0.25, 0.5, 1.0, 2.0 and 4.0 ppm of all element standard. The calibration curve of the blank was used to establish the limit of detection (LOD) and limit of quantification (LOQ) of the calibration method and presented typical values for ICP OES, according to Table 1, according to IUPAC recommendations [29]. These quantifications were carried out on the same day as the analysis (n = 3).

The accuracy of the data reported was ensured by recovering experiments performed according to Table 2. A certain amount of each element of interest was added before the mineralization of fruit samples, having an expected concentration in samples, which was evaluated by recovery. The analysis was performed in triplicate. Spike recoveries ranged from 88.98% to 109.17% with a relative standard deviation (%RSD) varying from 0.00% to 33.05%.

### 2.6. Comparison Criteria

The elemental concentration of each element in 100 g of fruit or 100 g of seed was compared to Dietary Reference Intakes (DRI), when available. DRI is the over-all term for reference values used to propose and evaluate nutrient intake in healthy people. Within DRIs recommendations, which varies regarding sex and age, there are three used references:

Recommended Dietary Allowance (RDA): average daily level intake to meet the requirements of a particular nutrient for about 97% to 98% of healthy people.

Adequate Intake (AI): it is a level of nutrient intake expected to guarantee nutritional adequacy when there is not sufficient data to define an RDA.

Tolerable Upper Intake Level (UL): the highest daily intake of a specific nutrient that adverse health effects are not likely to happen.

Taking that into account, we used the FDA criteria [30] for nutrient claims to determine if the pulp or the seed are sources for a specific element regarding its content.

### 2.7. Lipid Profile Determination

The extract of oil content occurred by soaking 100 g of separated parts of pulp and seed in 300 mL hexane for 14 days in a 500 mL glass bottle at room temperature. After this time, the solvent was concentrated in a rotary evaporator and further dried with a nitrogen flow. We converted the oil to fatty acid methyl ester (FAME) to determine its composition, according to the method described by Figueiredo et al. [31]. Triplicate analysis was performed; first, around 100 mg of each oil was added to 2 mL of 7% boron fluoride methanolic solution and 1 mL of toluene. This mixture was heated to 100 °C in the oven for 45 min and cooled down in sealed tubes until it settled to room temperature when we added water (5 mL), hexane (3 mL) and, sodium sulfate (300 mg). The preparation was stirred and left to settle, after which the top layer was collected and 1 µL injected into gas chromatography (GC) (model 6890 N; Agilent Technologies Inc., Santa Clara, CA, USA) to obtain individual peaks of FAME. The GC was equipped with a flame ionization detector and a polar capillary column (HP88, internal diameter 0.25 mm, length 100 m, film thickness 0.25 mm). The injector temperature was 225 °C, and the detector temperature 285 °C. The initial temperature condition for the column was at 160 °C, which was kept for 3 min and then further increased to 190 °C at 5 °C per minute for 6 min, then the temperature was augmented to 230 °C at 6 °C per min for 12 min. Individual FAME peaks were identified by comparing their relative retention times with the FAME standard (C8-C22, 99% pure; Supelco, Bellefonte, PA, USA). For additional control, samples were spiked with methyl undecanoate as an internal standard. Readings were analyzed using the Chemstation, version A09.01 (Agilent Technologies Inc., Santa Clara, CA, USA).

### 2.8. Thermogravimetric Analysis (TGA)

The oil of pulp and seed canjiqueira were evaluated for thermal stability in the TGA instrument, model Q50 (TA Instruments, New Castle, DE, USA). Proximate to 5 mg of sample was used, using platinum crucibles for support. The heating ramp set was 10 °C/min. The initial and final temperature was 25 °C, and 900 °C, respectively, in an air atmosphere with a purge flow of 60 mL/min in the oven.

### 2.9. Canjiqueira Pulp and Seed Extract Preparation and Compound Identification

Pulp and seed were submitted to extraction in an ultrasonic bath for 20 min using ethanol:water 7:3 (*v*/*v*) at the proportion of 1:4 fruit pulp or seed/solvent extract, twice. Then, the solvent was removed from the extract in a rotary evaporator and lyophilized. The pulp extract yielded 2.24%, and the seed extract produced 0.86%.

A solution at 1 mg/mL with ethanol and water (7:3 *v*/*v*) of the pulp and seed extracts was prepared, and 2 µL injected into a UFLC LC-20AD (Shimadzu) combined with a diode array detector (DAD), and a mass spectrometer microTOF-Q III (Bruker Daltonics) provided by an electrospray ionization source and analyzers quadrupole-time-of-flight (qTOF) (n = 3). A C-18 column (Kinetex, 2.6 μ, 150 × 2.2 mm) with the protection of a pre-column of the same material was used. Water (solvent A) and Acetonitrile (solvent B), were the mobile phase, both with 1% acetic acid (*v*/*v*). the gradient elution profile was 0–2 min 3% of B; 2–25 min 3–25% of B; 25–40 min 25–80% of B, then the column was washed and reconditioned for 8 min. The temperature of the column was kept at 50 °C, and the flow rate was 0.3 mL*/*min. The mass spectrometer operated in both negative and positive modes (m/z 120–1200), and we monitored the wavelength range from 204–800 nm.

We used UV spectra, accurate mass, and Electrospray ionization (ESI) fragmentation patterns to verify the compound identification and compared the results with those from previously published literature. The comparison among compounds was carried out using identified compounds of the fruit in our previous work, using the previously established run for reading [26]. *Trans-*piceatannol in pulp and seed extract quantification occurred as described by Santos et al. (2017) [26]. The calibration curve was built from the standard of *trans*-piceatannol, with the concentrations 0.39; 0.78; 1.56; 3.12; 6.25; 12.5; 25; 50; 100 and, 200 µg/mL. The quantification was performed in an HPLC-DAD with a reversed-phase C-18 monolithic column (Onyx/Phenomenex, 100 × 3 mm), with a pre-column of the same material. The mobile phases were deionized Water (phase A) and Acetonitrile (phase B), with an isocratic elution profile with 30% of B from zero to 16 min. The flow rate was 1.2 mL/min.

The UFLC-TOF was used to confirm the compound by comparing the results of retention and fragmentation times with the authentic standard injected in the same equipment. Minor compounds were not identified once they did not show fragmentation.

### 2.10. Statistical Analysis

GraphPad Prism 8.4.1 was used to run the statistical analysis with a two-tailed Student’s *t*-test in the mineral content and fatty acid profile to compare the mean values [32] for data with parametric distribution, while for non-parametric distribution, the Mann–Whitney post-test was used. A significant difference was determined when *p* < 0.05.

## 3. Results and Discussion

### 3.1. Mineral Content

We compared the elemental concentration to the DRIs (RDA, AI, and UL) regarding their amount using the FDA criteria for nutrient claims. The DRIs are a reliable, comparable source, used to evaluate if diets deliver enough nutrients to meet necessities not being excessive [33].

The mineral content in seed and pulp of canjiqueira, relative amounts with DRI, and statistical analysis of seed and pulp are presented in Table 3. The seed and the pulp canjiqueira are rich in calcium (Ca) concerning the RDA references (USDA, 2006), not being toxic for any gender and age groups, with the seed being more abundant in this element. However, there were no statistically significant differences between seed and pulp (*p* = 0.6667). Kumssa et al. (2015) [34] assessed calcium deficiency worldwide and found that, in 2011, there were 3.5 billion people with some level of insufficiency. Ca supply was 644 ± 3 mg/day at a global scale, being Africa the continent with lower ingestion levels. The consumption of Ca-rich foods could diminish Ca deficiency.

Copper (Cu) concentration ranged from 0.3 mg/100 g in pulp to 0.78 mg/100 g in the seed; both could be considered an excellent source of this element, with the seed being statistically more abundant in this element (*p* = 0.0057). Cu is an essential element needed in several functions such as catalytic cofactor as a part of the structural constituent for proteins, with roles in vital biological processes, as enzyme activity, oxygen transport, and cell signaling [35].

Iron (Fe) is found in an excellent amount (pulp 1.89 mg/100 g; seed 4.24 mg/100 g, with no significant results when comparing the two fruit parts, (*p =* 0.0527)), but not high enough to cause toxicity once the concentration of Fe does not reach the UL. Fe deficiency is the most common nutritional deficiency. Globally, a high proportion of anemia is caused by Fe deficiency, reaching nearly 64% in central Asia, 54% in South Asia, and 62% in Andean Latin America [34,36]. Fruits and byproducts such as canjiqueira seeds, which are very concentrated in Fe, should be considered as strategies to tackle anemia.

Both pulp and seed are an excellent source of potassium (K) (1507.15 mg/100 and 891.16 mg/100 g respectively), with the concentration reaching over 50% of adequate intake (AI) in the pulp, with the pulp baring higher amounts of this element (*p =* 0.0334). The excess of K above the AI is readily excreted in the urine of healthy people, and there is no indication that a high intake of K from foods has adverse effects in this population (WHO, 1996). In this way, the elevated concentration of K in canjiqueira should not be a general health concern.

Pulp (143.24 mg/100 g) and seed (151.90 mg/100 g) are excellent sources in magnesium (Mg) regarding the RDA, where the results did not differ (*p* > 0.9999). Mg plays a pivotal role in health in the physiological brain, heart, and skeletal muscle functions [37]. Other than that, Mg supplementation correlates with antihypertensive effects [38].

The concentration of manganese (Mn) is also higher in seed (0.90 mg/kg) than pulp (0.63 mg/100 g). Still, the results do not show any statistical difference between pulp and seed (*p =* 0.0575), both being excellent sources in this mineral, and neither of them presenting a toxicity risk concerning UL values. Mn is essential to human life, required in the action of an assorted series of enzymatic proteins (e.g., arginase and glutamine synthase) [39].

Neither seed nor pulp is considered good sources of sodium (Na) about AI, with results showing no difference between these fruit parts (*p* = 0.3333), and these low levels are typical in plants [40].

Both selenium deficiency and excess are related to some health impairments. While low selenium intake correlates to viral virulence, prostate cancer, higher mortality risk, and autoimmune thyroid dysfunctions, the excess of this mineral is related to type 2 diabetes risk, prostate cancer risk, alopecia, dermatitis, and mortality risk [41,42]. The insufficiency of selenium is more abundant. However, the prolonged exposure to high doses of selenium intake, i.e., superior to 200 µg/day, is related to these outcomes [41]. Usually, this elevated intake is from the supplementation of selenium. Nonetheless, both pulp and seed of canjiqueira have a high amount of selenium (160 and 180 µg/100 g, respectively, which do not display statistical difference (*p =* 0.3349). In this sense, the chronic use of this fruit parts should be monitored.

Only the seed can be considered a good source of zinc, while the pulp is not rich in this element. Neither of the parts is a risk for acute toxicity. This raw number somewhat may affect its classification regarding the DRIs, but it does not present a statistical relevance (*p =* 0.3333). Zinc is a crucial element to cell integrity, participating in the process of cell division, growth, and development [43]. The most prominent adverse effects of zinc deficiency are the diminished cell-mediated immunity, impairment of cognitive functions, increased oxidative stress, and the upregulation of inflammatory cytokines [44].

Aluminum, cobalt, and molybdenum were below the LODs. The accumulation of metals in plants may derive from several environments, like soils with innately elevated metal concentration [45] or soils contaminated by processes such as mining or smelting, the use of pesticides and fertilizers, as well of the wrongly discard of electronic devices [46,47,48].

### 3.2. Fatty Acids Profile

Table 4 presents the differences between the fatty acid (FA) composition of pulp and seed. Twelve fatty acids were identified, in which saturated fatty acid (SFA) concentration was 37.1% in pulp and 24.6% in the seed.

Saturated fatty acid intake is related to increase blood concentration of LDL-c, a typical example of dyslipidemia [49,50], which is an established risk factor for ischemic heart diseases. However, different saturated fatty acids appear to act at different intensities in this lipidemic context. The myristic acid (C14:0) is more powerful to raise the levels of LDL, followed by lauric (C12:0) and palmitic acids (C16:0) [51]. The main SFA in both samples was palmitic acid, which in the pulp, this FA accounted for 30.7%, while in seed, it represented 18.2% of total FA. These three FA are present in higher amounts in the oil of the pulp, rather than the seed oil, presenting a significative difference in the sum of all of these fatty acids (0.0086 for lauric acid, 0.0001 for myristic acid and <0.0001 for palmitic acid). These data show that the seed oil of canjiqueira could be less prejudicial than canjiqueira pulp oil.

The oil of both pulp and seed presents a high concentration of oleic acid, the lead monounsaturated fatty acid (MUFA), with the pulp having 47.3% and the seed 39.3% (*p <* 0.0001). The MUFA content is very close in both parts of fruit, with prominent concentrations in seed (40.3%) and pulp (49.2%), but the latter presents a higher value. The MUFA concentration is lower than olive seed (70.32%) and pulp (61.82%) [52]. Nevertheless, while canjiqueira pulp presents fewer oleic acid than avocado pulp with 64.43%, known for its high oleic acid concentration, the seed of canjiqueira is more abundant in oleic acid than avocado seed with 17.41% [53].

The MUFAs role in health is primarily associated with oleic acid, which includes lowering blood pressure [54], and that may improve insulin sensitivity and glucose control [55]. In addition to other protective effects, such as lower low-density lipoprotein (LDL) and total triacylglycerol (TAG) levels, it can increase the high-density lipoprotein (HDL) levels [56,57]. However, the effects of oleic acid often are observed in substitution of SFA, which is related to dyslipidemias. Hence, the results are not evident yet [54]. Furthermore, the most studied MUFA is the one in olive oil, and several health benefits related to its intake is due to the distinctive blend of phenols presented in the olive oil, being the phenols the most bioactive resident compounds [58].

Seed only showed a high concentration of PUFA, represented by 31.9% of total FA in seed, while the pulp exhibited just 9.0% of this FA class. The major PUFA was linoleic acid (LA) in pulp (8.5%) and seed (31.7%), differing greatly in LA content (*p* < 0.0001). These results concur with those described by Berto et al. (2015) [27] in Amazonian fruits, with LA being the most predominant PUFA in pulp and seeds in various fruits. Linoleic acid (18:2 n-6) is the main family compound known as n-6 PUFAs, which can be elongated and desaturated to other n-6 series bioactive PUFAs such as γ-linolenic acid (18: 3 n-6) and arachidonic acid (AA) (20: 4n-6) [59]. Sequentially, arachidonic acid can transform into a legion of bioactive compounds named eicosanoids, such as prostaglandins and leukotrienes, considered to be essential for the metabolic maintenance of cells membranes and tissues when consumed in adequate amounts [59,60].

Although this metabolic path is viable, the elevated consumption of PUFA and PUFA rich oils related to plasma α-linolenic acid (ALA), but not plasma AA. On the other hand, meat intake favored plasma AA, which indicates that the pre-formed AA ingestion is more determinant to plasma AA than LA intake [61]. These data corroborate the advantage of seed oil over the pulp oil.

### 3.3. Thermal Stability

The TGA was selected to evaluate the canjiqueira oil stability once it is a research tool to assess the resistance of fats and oils to oxidation and then to predict the beginning of oxidation more quickly in comparison with the traditional oven aging, that could take weeks or months [62].

From an analytical and technological point of view, the high occurrence of SFA is related to higher oxidative lipid stability, since the presence of unsaturation, especially of PUFAs are more susceptible to oxidative processes [63]. The oxidation process is related to the arise of off-flavors due to the deterioration of volatile compounds [64].

**Figure 1:**
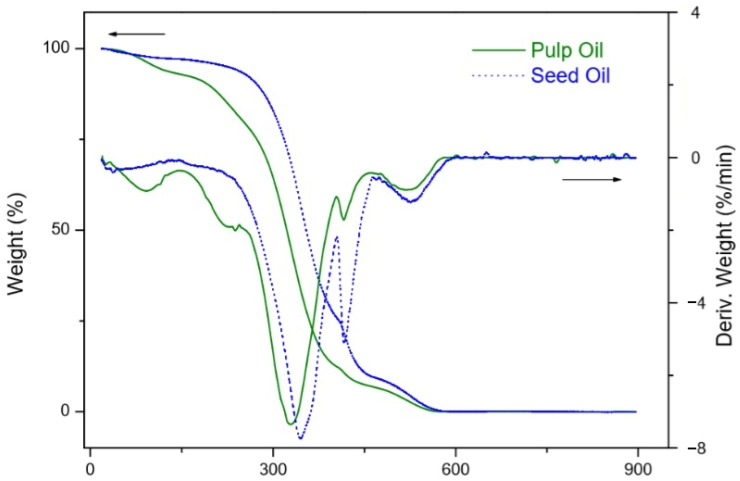
Thermal stability canjiqueira pulp and seed oils.

However, even pulp oil presents a higher amount of saturated fatty acids than seed oil; the seed oil was more stable than pulp oil (Figure 1). According to the TGA method, the later the development of mass loss, the higher the stability of the oil [65].

The characteristics of edible oils are affected by the presence and concentration of various trace metals, which are used to evaluate their quality. Trace metals as Cu, Zn, Fe, Mn, and Ni can augment oil oxidation [66]. Thus, the presence of polyvalent metals as Fe and Cu makes the oil less stable to oxidation [67]. Even though the seed is more concentrated in both of these elements, the seed oil is more resistant to oxidation, regardless of the presence of polyvalent transitional metals and unsaturations.

**Figure 2:**
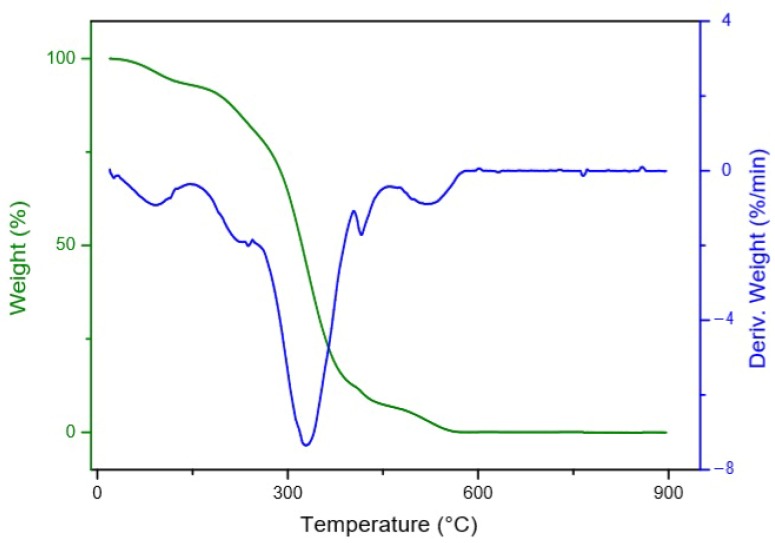
Thermal decomposition of canjiqueira pulp oil.

**Figure 3:**
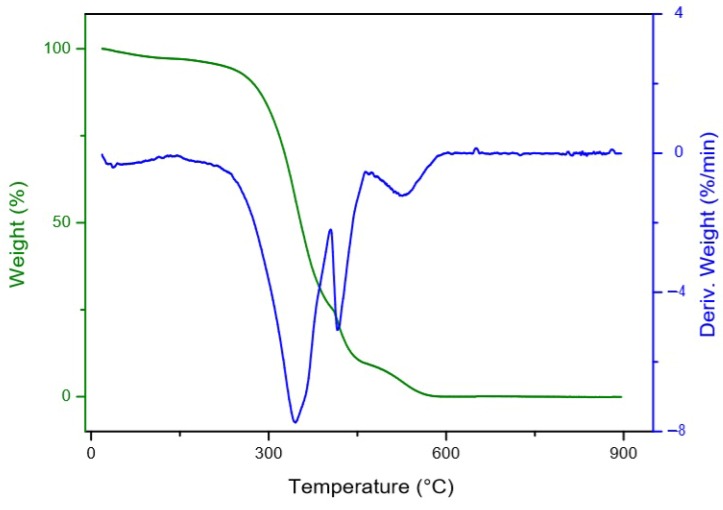
Thermal decomposition of canjiqueira seed oil.

Pulp oil showed four main thermal decomposition steps in the thermogravimetry/differential thermogravimetry (TG/DTG) curves (Figure 2), while the seed oil showed three significant thermal decomposition steps (Figure 3). The first two steps for the pulp oil and the first step for the seed oil, with an onset temperature of thermal decomposition between 100–200 °C can be related to moisture loss and the volatilization of aldehydes, ketones and short-chain fatty acids [68].

The use of antioxidants in edible oils has the purpose of protecting unsaturated fatty acids, enhancing the stability of thermal degradation that commonly happens at a temperature range of 150–220 °C [69].

Canjiqueira pulp oil and canjiqueira seed oil showed similar thermal stability to other native Brazilian fruits, such as Babassu, which has a very saturated profile [70]. Besides that, the oils from both pulp and seed presented higher thermal stability than conventional culinary oils, such as soybean (SB) oil and sunflower (SF) oil [71]. In this way, the thermal stability of canjiqueira seed oil may relate to the presence of natural antioxidants. The same phenomena occurred in corn oil analyzed by Santos et al. (2002) [72], which attributed the corn oil stability to tocopherol and ferulic acid, besides the presence of artificial antioxidants. The addition or presence of antioxidants in frying oils minimized the postprandial oxidative stress and the succeeding DNA impairment in people that consumed the oil when compared to the harmful use of SF oil [73].

Marcelino et al. (2019) [24] reported high antioxidant capacity in ripe whole fruits of *B. cydoniifolia* assessed by DPPH (IC_50_ 0.11 mg/L) and ORAC methods (78.6 µmol TE/kg) and could explain the oil stability. Additionally, we found a higher concentration of piceatannol in the seed than the pulp (presented on Section 3.4), corroborating with the results of thermal stability. The antioxidant activity presented by Marcelino et al. (2019) is superior to those found for red grape skins, that concentrates most of the fruit resveratrol [74], and passion fruit seeds [75], known for its piceatannol concentration.

When considering the effects of SFA intake in health and that the reduction of its consumption in favor of unsaturated fatty acids could lower the risk of cardiovascular events in about 14% [76]. The higher thermal stability of canjiqueira seed oil in comparison to canjiqueira pulp oil could help the culinary use of unsaturated oils rather than saturated fat without producing higher health risks or the production of off-flavors.

### 3.4. Piceatannol in B. cydoniifolia Pulp and Seed

**Figure 4:**
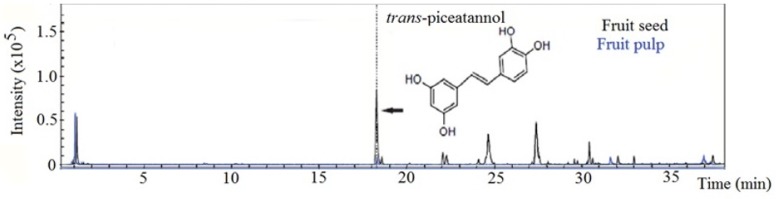
Chromatography profile of *Byrsonima cydoniifolia* seed and pulp extracts.

Several compounds of interest have been identified in canjiqueira pulp, with application in industry and health. Antioxidants as quinic acid, resveratrol, and quercetin are present in canjiqueira pulp. Besides that, *trans*-piceatannol was considered the main component in the pulp extract [26], with a higher concentration than in species that knowingly accumulate such compounds, as it occurs in grape varieties [77]. Although *trans*-piceatannol was identified in canjiqueira pulp, to the best of our knowledge, this is the first time that this compound is verified in canjiqueira seed. We choose to emphasize *trans-*piceatannol only, once it is the crucial antioxidant compound identified in our previous study in the pulp, and now in the seed (Figure 4).

As it occurs in other species, such as passion fruit [78], piceatannol is present in seed in higher amounts than in the pulp. Figure 4 shows the *trans-*piceatannol concentration of the pulp (2.085 μg/mg) and the seed (127.315 μg/mg) extracts. As well, piceatannol was quantified on the outer seed integument, with no difference regarding the inner seed (Supplementary Material).

Piceatannol effects on human health include improved insulin sensitivity, lower blood pressure, and heart rate in overweight men [79]. The supplementation of piceatannol in animals fed a high-fat diet showed a hypolipidemic effect, lowering total cholesterol, LDL cholesterol and atherogenic index [80], and also anti-obesity [81] when compared to animals in the same diet without piceatannol supplementation.

Many of the health effects that are displayed by piceatannol are related to its antioxidant activity, which, in turn, is higher than its parent, resveratrol [82]. In this sense, the seed of canjiqueira could be more beneficial to health than the pulp itself.

## 4. Conclusions

Mineral profiles of both pulp and seed are rich in different elements. While the pulp is more abundant in K and Na, the seed is more concentrated in Ca, Cu, Fe, Mg, Mn, Se, and Zn. Pulp and seed have a very unsaturated fatty acid profile, and both have oleic acid as their major MUFA and linoleic acid as the principal PUFA. Pulp and seed of canjiqueira are significant sources of essential fatty acid, in this case, linoleic acid, in addition to revealing its potential nutritional and health benefits.

The thermal stability of seed oil was superior to pulp oil, regardless of the presence of more unsaturations on the fatty acid profile and the presence of polyvalent metals. The thermal stability of seed oil was attributed to the presence of natural antioxidants, which was demonstrated by the seed higher concentration of piceatannol than pulp.

Although the pulp is commonly used as a source of nutrients, and product manufacturing, the seeds of canjiqueira are discarded. This study showed that both parts of the canjiqueira fruit could be used as a strategy to address nutrition issues and are valuable ingredients to prospective food products. Besides that, it is imperative to conduct further research to prove canjiqueira potential value, comparing its nutritional characteristics with other fruits and oils, and ensuring its safe consumption by determining heavy metals; and conducting in vivo studies.

## Figures and Tables

**Table 1:** Parameters of calibration curves obtained by external calibration, correlation coefficient (R^2^), the limit of detection (LOD) and limit of quantification (LOQ) by using ICP OES.

Analyte	R^2^	Equation External Calibration	LOD mg/kg	LOQ mg/kg
Al	0.9996	y = 457.76x + 8.7836	0.056	0.187
Ca	0.9993	y = 70225x + 1953.4	0.003	0.011
Co	0.9998	y = 4161.7x + 5.8914	0.003	0.008
Cu	0.9997	y = 13021x + 107.6	0.003	0.010
Fe	0.9996	y = 3451.9x + 31.784	0.007	0.022
K	0.9969	y = 290.72x + 18.667	0.345	1.149
Mg	0.9997	y =284979x + 3438.5	0.001	0.002
Mo	0.9998	y = 2201.2x + 4.468	0.001	0.005
Mn	0.9999	y = 37724x + 281.01	0.0004	0.001
Na	0.9995	y = 1167.7x + 7.7392	0.086	0.287
Se	0.9999	y = 365.94x + 1.7592	0.011	0.037
Zn	0.9999	y = 8849.5x + 74.424	0.0005	0.002

Calibration equations (y = ax + I), y = intensity; a = slope; x = concentration (mg/kg); I = intercept.

**Table 2:** Addition and recovery tests to verify method precision for all analytes obtained in ICP-OES (n = 3).

Analyte	Accuracy			Precision
	Expected Value mg/L	Obtained Value mg/L	% Recovery	% RSD
Al	1.00	1.09 ± 0.003	109.17	0.27
Ca	1.00	0.89 ± 0.139	88.98	15.61
Co	1.00	0.95 ± 0.000	94.75	0.00
Cu	1.00	1.08 ± 0.003	108.28	0.27
Fe	1.00	1.11 ± 0.001	110.98	0.09
K	1.00	1.18 ± 0.390	118.50	33.05
Mg	1.00	0.90 ± 0.069	90.13	7.66
Mo	1.00	1.01 ± 0.001	101.29	0.10
Mn	1.00	0.94 ± 0.016	94.04	1.70
Na	1.00	1.09 ± 0.036	109.54	3.30
Se	1.00	0.90 ± 0.002	89.79	0.22
Zn	1.00	0.93 ± 0.001	92.94	0.10

Results expressed as average ± SD.

**Table 3:** Mineral content in pulp and seed of canjiqueira mg/100 g (dry weight).

Canjiqueira mg/100 g						
Element	Pulp	% Regarding DRIs	Seed	% Regarding DRIs	DRI (RDA/AI *)	* p
Al	<LOD	NA	<LOD	NA	NA	NA
Ca	295.56 ± 3.92	24.63–29.56	381.36 ± 3.92	31.78–38.14	1000–1200	0.6667 ^†^
Co	<LOD	NA	<LOD	NA	NA	NA
Cu	0.30 ± 0.02	33.33	0.78 ± 0.05	86.66	0.9	0.0057
Fe	1.89 ± 0.09	10.50–23.63	4.24 ± 0.79	23.56–53	8–18	0.0527
K *	1507.15 ± 100.70	44.33–57.97	891.16 ± 128.67	26.21–34.28	2600–3400 *	0.0334
Mg	143.24 ± 0.40	34.10–46.21	151.90 ± 32.44	36.17–49	310–420	>0.9999 ^†^
Mo	<LOD	NA	<LOD	NA	NA	NA
Mn	0.63 ± 0.04	27.39–35.00	0.90 ± 0.09	39.13–50	1.8–2.3	0.0575
Na *	1.08 ± 0.13	0.07	0.74 ± 0.19	0.05	1500 *	0.3333 ^†^
Se	0.16 ± 0.03	NA	0.18 ± 0.01	NA	NA	0.3349
Zn	0.48 ± 0.04	4.36–6	1.28 ± 0.31	11.64–16	8–11	0.3333 ^†^

LOD—elemental contents were below the limits of detection. NA—not applicable (no DRI available or amount detected not significant for DRI). * *p* values determined by the Student *t*-test represent a difference between pulp and seed means when lower than <0.05. ^†^ Mann–Whitney test.

**Table 4:** Fatty acid distribution (%) in pulp and seed of *B. cydoniifolia.*

Fatty Acid	Pulp	Seed	* p
**Saturated fatty acids**			
C10:0 –Capric acid	0.2 ± 0.02	0.1 ± 0.01	0.0015
C12:0 –Lauric acid	0.4 ± 0.03	0.3 ± 0.02	0.0086
C14:0 –Miristic acid	0.6 ± 0.02	0.4 ± 0.02	0.0001
C16:0 –Palmitic acid	30.8 ± 0.31	18.2 ± 0.34	<0.0001
C18:0 –Estearic acid	4.2 ± 0.04	4.9 ± 0.10	0.0004
C20:0 –Araquidonic acid	0.8 ± 0.03	0.5 ± 0.04	0.0005
C22:0 –Behenic acid	0.2 ± 0.01	0.1 ± 0.02	0.0015
Total (%)	37.1	24.5	
**Monounsaturated fatty acids**			
C16:1 –Palmitoleic acid	1.4 ± 0.06	0.65 ± 0.02	<0.0001
C18:1 –Oleic acid	47.6 ± 0.35	39.3 ± 0.30	<0.0001
C20:1 - Cis-11-Eicosenoic acid	0.2 ± 0.01	0.3 ± 0.01	0.0003
Total (%)	49.2	40.3	
**Polyunsaturated** **fatty acids**			
C18:2 –Linoleic acid	8.5 ± 0.00	31.7 ± 0.11	<0.0001
C18:3n3 –Linolenic acid	0.5 ± 0.01	0.2 ± 0.01	<0.0001
Total (%)	9.0	31.9	

Results expressed as average ± SD. * *p* values determined by the Student *t*-test represent a difference between pulp and seed when lower than <0.05.

## Supplementary Materials

The following are available online at https://www.mdpi.com/2304-8158/9/4/521/s1. Table S1. Operating conditions for the microwave digestion system. Table S2. Operation conditions of ICP OES. Figure S1. Piceatannol quantification in *Byrsonima cydoniifolia* (canjiqueira).
