# Supplementary material for: Canjiqueira Fruit: Are We Losing the Best of It?
Source: Foods. 2020 Apr 21;9(4):521. doi: 10.3390/foods9040521 (PMC7231018; doi:10.3390/foods9040521)

Table S1. Operating conditions for the microwave digestion system

Stage	Temperature/°C	Ramp time/min	Hold time/min	Power/W	Pressure (bar)
1	145	2	5	1305	40
2	170	5	10	1305	40
3	190	2	15	1305	40
4	50	1	10	0	0

Table S2. Operation conditions of ICP OES

Parameter (Unit)	
RF Power (W)	1250
Sample uptake rate (L min^{-1})	0.5
Plasma gas flow rate (L min^{-1})	12
Integration time (s)	5
Stabilization time (s)	20
Nebulizer flow (psi)	30
Plasma Viewing Mode	Axial
Element	Wavelength (nm)
Al	396.152
Ca	422.673
Co	238.892
Cr	425.435
Cu	327.396
Fe	259.940
K	769.896
Mg	285.213
Mn	257.610
Na	589.592
Ni	221.647
P	185.942
S	180.731
Se	196.090
Zn	213.856

Figure S1. Piceatannol quantification in *Byrsonima cydoniifolia* (canjiqueira)

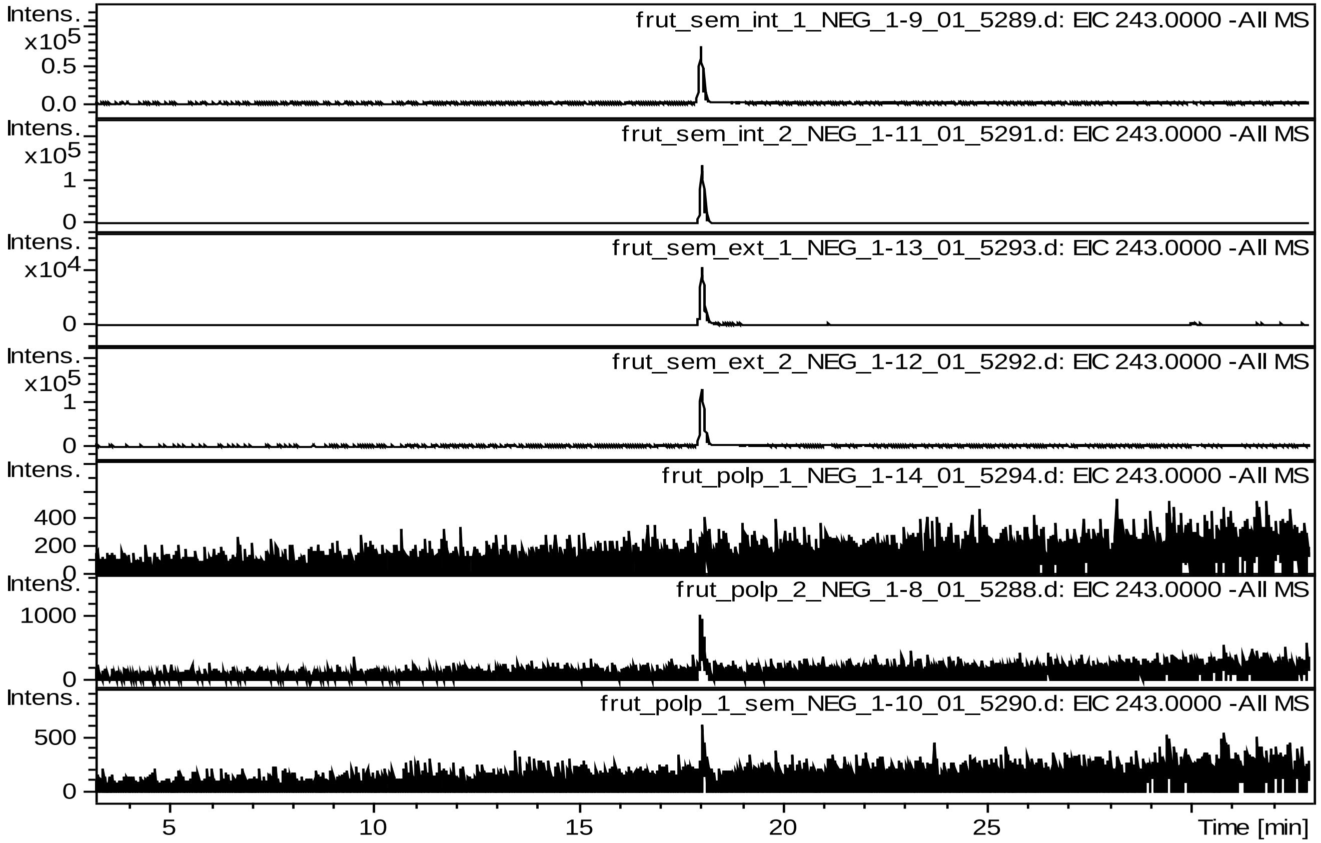

1
Inner seed_1: 72.189
Inner seed_2: 127.315

2
Outer seed_1: 51.910
Outer seed_2: 126.106

3
pulp_1: 0
pulp_2: 2.085

1: first extraction; 2: second extraction

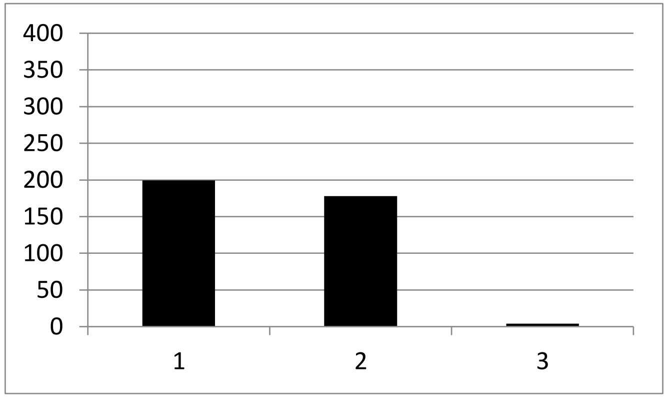

Supplement: Supplementary file 1 [file foods-09-00521-s001.pdf]
